# Crowd counting in domain generalization based on multi-scale attention and hierarchy level enhancement

**DOI:** 10.1038/s41598-024-83725-5

**Published:** 2025-01-02

**Authors:** Jiarui Zhou, Jianming Zhang, Yan Gui

**Affiliations:** School of Computer and Communication Engineering, Changsha University of Science and Technology, Changsha, 410114 China

**Keywords:** Crowd counting, Spatial attention, Channel attention, Multi-scale features, Domain generalization, Computer science, Information technology, Scientific data

## Abstract

In order to solve the problem of weak single domain generalization ability in existing crowd counting methods, this study proposes a new crowd counting framework called Multi-scale Attention and Hierarchy level Enhancement (MAHE). Firstly, the model can focus on both the detailed features and the macro information of structural position changes through the fusion of channel attention and spatial attention. Secondly, the addition of multi-head attention feature module facilitates the model’s capacity to effectively capture complex dependency relationships between sequence elements. In addition, the three-stage encoding and decoding processing mode enables the model to effectively represent crowd density information. Finally, the fusion of multi-scale features derived from different receptive fields is further enhanced through multi-scale hierarchy level feature fusion, thereby enabling the model to learn high-level semantic information and low-level multi-scale visual field feature information. This method enhances the model’s capacity to capture key feature information, even in highly differentiated datasets, thereby improving the model’s generalization ability on a single domain. The model has demonstrated strong generalization capabilities through extensive experiments on different datasets. This study not only improves the accuracy of crowd counting, but also introduces a new research approach for single domain generalization of crowd counting.

## Introduction

The importance of crowd counting research has been highlighted with the acceleration of urbanization construction, and some research works^1^ have shown effectiveness in specific fields in practical application scenarios. In recent years, the acceleration of urbanization and the frequent holding of large-scale public events have highlighted the growing importance of accurately and efficiently counting crowds. However, for scenes with drastic scale changes, inconsistent density, or complex backgrounds, crowd counting remains a challenging problem. Therefore, many studies are still dedicated to overcoming these challenges. At present, the majority of existing advanced methods use density maps^2–4^ as targets and use convolutional neural networks (CNN) for model inference^5–7^, while others use detection methods^8,8^ for crowd counting.

**Fig. 1:**
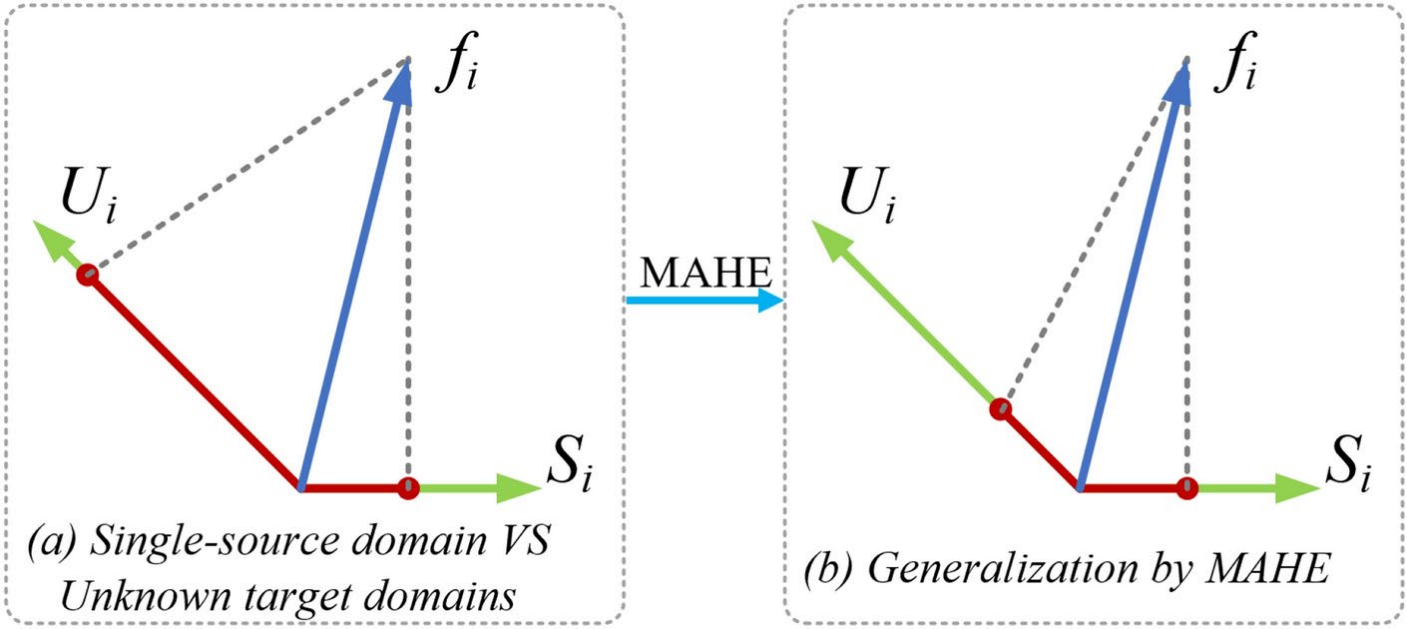
In single domain and unknown target domain (**a**) and Single domain generalization improvement (**b**).

A significant challenge in crowd counting comes from the scale changes caused by perspective distortion between different images and within a single image^9^. This perspective change causes individuals in the image to appear larger in scale up close, while becoming very small in the distance, resulting in significant scale differences in the dataset^10^. This difference not only increases the difficulty of object detection, but also poses higher requirements for subsequent counting tasks, requiring algorithms to robustly handle and adapt to crowd counting at different scales. Directly merging multi-scale branches alone cannot effectively handle the performance differences of each pixel on multi-scale density maps. Using density experts’ hierarchical mixing to redesign multi-scale neural networks^11^, which hierarchically merge multi-scale density maps and perform crowd counting, is an effective approach. Inspired by these ideas, we propose a scheme for a multi-scale feature fusion at different levels, which enhances the adaptability of the model to crowds of different scales.

The existing methods for crowd counting are mainly trained and tested in similar scenarios. When the model’s testing scenario is different from its training scenario type, the performance of crowd counting methods is significantly reduced^12^. The main reason is that the crowd scenarios vary greatly and lack the capacity for generalization across different data domains, which seriously hinders the widespread application of crowd counting. The method of knowledge transfer and complementarity between multiple domains^13^ has been preliminarily validated, and the improved dual source knowledge mixing enhancement strategy can enhance the model’s adaptability. The main difficulty lies in the large domain gap between complex real-world datasets, where images vary greatly in style, density level, and content^14^.

To address the above issues, this paper proposes a new multi-scale attention and hierarchy level enhancement (MAHE) scheme. The primary objective is to improve the model’s generalization capacity within a single domain by utilizing multi-scale attention and hierarchy level enhancement methods. Our contributions can be summarized as follows:

(1) By employing channel feature enhancement techniques, MAHE enhances the robustness of the model and effectively reduces prediction errors.

(2) The use of spatial attention feature enhancement improves the feature description of the model at any spatial position.

(3) By integrating multi-scale features, the model’s capacity to classify crowds of different scales has been enhanced.

(4) The hierarchy level fusion mechanism effectively activates low-level features and improves the feature expression capability of the encoding layer.

By integrating these innovative points, the framework of this study not only optimizes the accuracy of model inference calculations, but also improves the robustness of crowd counting in complex scenarios. The experimental results show that traditional methods for training models on single source domain data have large gaps in the partitioning of unknown target domains, as shown in Fig.1a, while the proposed method MAHE has a smaller interval in the unknown target domain, indicating good compatibility with MAHE, as shown in Fig.1b. Here, *i* is the number of the test sample, $S_i$ represents the source domain, $U_i$ represents the unknown target domain, and $f_i$ represents the feature projection of the data domain established by the model. We hope that the results of this study can provide a new perspective for the field of population counting and provide valuable references for future research.

## Related work

We briefly reviewed recent work on crowd counting, attention mechanism, multi-scale features, and domain generalization.

### Crowd counting

Since the adoption of the VGG network^15^, there has been a significant advancement in the field of crowd counting. Crowd counting is a fundamental tool for the understanding pedestrian patterns and the analysis of crowd flow. Counting the total number of crowd in a given video without relying on tracking poses a significant challenge in terms of efficiency. Using end-to-end decomposition and inference networks and density estimation methods to predict the initial number of crowd has been proven feasible^16^. Designing effective deep learning architectures can alleviate the issue of data localization in specific dense scenarios. Toha et al. used crowd localization maps to accurately and effectively locate and count participants in dense crowds^17^, and improved the accuracy and efficiency of the model through the concepts of residual layers and dilated convolutions. However, generating priority maps based on feedback from perception blocks in the receptive field can predict the degree of crowding in crowded areas^18^. It is challenging to obtain crowd counts that overly rely on position level annotations in real-world situations. When only weak monitoring signals at the count level are available, using a multi granularity MLP regressor to regress the total crowd count can achieve better results than existing weakly supervised counting algorithms^19^. Due to the difference in shooting angles, the crowd counting model produces different output results. It is necessary to consider how to obtain stable crowd counting results with a reduced number of camera inputs. Using the expectation maximization algorithm to learn the prototype of foreground and density from limited supporting images and guiding the crowd counting of query images from local to global perspectives^20^ shows that it is effective. When the scale changes dramatically and the background noise interference is large, it will seriously affect the counting accuracy. In addition to using the original image information, recent studies have employed heat source information to address the inadequate representation of RGB features in low-light conditions. Kong et al. designed a Transformer based multimodal mixer^21^ to fully fuse the features of the two modalities, and then used a Transformer based network to replace the multi-head self-attention layer with an average pooling layer, constructing a dual-flow backbone network, and extracting rich multi-scale feature information from the two modalities.

The manual annotation of crowd counting training datasets has always been a challenging task, and recent advancements have been made in this area. Rita et al.^22^ developed a method for automatically constructing synthetic image training sets for specific scenes, eliminating the need for end users to manually annotate or collect representative images of the target scene. This approach offers a reliable reference for the fine-tuning of training data models. When there are significant differences in observation areas, using complex image area management and graphic erasure can help the model identify and process areas with high prediction difficulty^23^. By adjusting the prediction results of these areas, more accurate density maps can be generated. Jiang et al.^24^ obtained initial features through a basic network, enhanced these features through a class independent mask module, and used a class increment module to learn new knowledge while maintaining existing knowledge. Finally, the entire model was optimized through different loss functions to improve the accuracy and efficiency of crowd counting. Wang et al.^25^ promoted knowledge transfer from teacher models to student models from a different perspective. Student models can gradually learn and imitate the feature representations, mapping relationships, and predictive capabilities of teacher models, while maintaining accurate predictions of real data.

### Attention mechanism

The inspiration for the attention mechanism can be found in the unique selective attention capacity of the human visual system, which quickly promotes the progress and development of image processing tasks. The attention mechanism not only enhances the model’s capacity to obtain key feature information from crowd images, but also significantly improves the accuracy and efficiency of the model in handling complex crowd analysis tasks. Meng et al.^26^ developed an adaptive assisted learning task learning method based on graph structure, which constructs an attention enhanced adaptive shared backbone network that can simultaneously learn task sharing and task customization features, and train them in an end-to-end manner. Zhou et al.^27^ proposed a complementary attention enhanced MC3Net network architecture, with ConvNext^28^ as the backbone, to predict crowd density maps through different modules. For cross modal model design, Liu et al. proposed the CCANet network structure, which mainly consists of the Collaborative Cross modal Attention Module (CCAM) and the Collaborative Cross modal Fusion Module (CCFM)^29^, to fully capture complementary features in different modalities. Different lighting conditions and changes in weather conditions will also affect the model. For example, changes in rainstorm or fog can significantly affect the image quality, thus affecting the accuracy of crowd counting^30^. For the dynamic changes in the scene, the adaptability of the crowd counting model needs more attention. A Segmented Guided Attention Network (SGANet) with Inception-v3 as the backbone^31^ effectively expands the boundaries of this work.

Separating the foreground and background of the research object to improve the performance of the model has also attracted the attention of researchers. Ling et al.’s ConvNeXt based foreground estimation module extracts motion features from bidirectional frame differences and outputs a foreground estimation map, which is converted into attention weights^32^ for crowd counting to achieve good performance. Considering the importance of global and local views from a more comprehensive perspective of contextual information, Wang et al. proposed a new context attention fusion network, abbreviated as CAFNet^33^, for crowd counting. It utilizes cross level context and designs a guided attention fusion module to fuse low-level feature maps into high-level contextual information guidance, effectively compensating for spatial detail information. Zhang et al.^34^ integrated non-local attention mechanisms into inter-layer and intra-layer attention, respectively extending the receptive field to the entire image within the same layer and different layers, thereby capturing long-range dependencies and effectively overcoming huge changes in the scene.

### Multi-scale features

The problem of large-scale differences in crowd counting is one of the important challenges in this field, which seriously affects the accuracy and stability of crowd counting. Some researches have improved the flexibility of discrete scales by modeling continuous scale changes^35,36^ and using appropriate dilation kernels to adapt to different spatial positions. The traditional multi-scale neural networks exhibit larger scales for targets in the near range and become very small in the far range, which presents a significant challenge in meeting the task of crowd counting with significant scale differences.^37^. Jiang et al.^38^ achieved significant results by aggregating multi-scale views and combining Compare and Focus strategies to address the impact of redundant information and the problem of an imbalanced density distribution. Zhai et al.^39^ proposed a local multi-scale global contextual spatiotemporal network for gait recognition, which extracts multi-scale temporal features through subsequences with multiple spatial resolutions and achieves good results. How to capture changes in scale and quickly monitor the impact of scale differences on subsequent results, a scale sensitive crowd density map estimation framework provides a reference idea [11]. This framework can learn target scale changes, and the size of its Gaussian kernel adapts to the size of the target^40^. The deformable density map decoder (DDMD) introduces deformable convolutions to adapt to changes in the Gaussian kernel and improve the scale sensitivity of the model. It also uses selective inheritance learning to find the most suitable object scale to enhance feature extraction^41^.

Sometimes perspective distortion in a scene can pose greater challenges, often involving collaborative learning with multiple resolutions and angles. It is very difficult to obtain the most discriminative features from each metric learning. Jiang et al.^42^ proposed a dedicated feature selection and inheritance adapter that selectively forwards scaled custom features at each scale, as well as a masking selection and inheritance loss that helps to achieve high-quality density maps at all scales. As the scene size changes and the background noise increases, even optimized features may be difficult to effectively estimate crowd information. A new framework called Multi Path Scaling Network (MZNet)^43^ uses multiple scaling paths to recursively optimize multi-scale features and gradually enhance foreground information to improve crowd counting performance. Recently, approaches have been developed to accurately enhance attention to the head region and reduce misjudgment of the background region. These approaches begin with hierarchical scale calibration and the Spatial Attention Network (HSNet)^44^.

### Domain generalization

In practical tasks of crowd counting, we often encounter the same problem where a model trained on one dataset performs poorly on another dataset. When the scene and camera layout change, it causes the cross-view data domain changes. Through the multi-view crowd counting paradigm^45^, and training and testing crowd counting tasks in different scenes with arbitrary camera layouts, it has good performance, however, this method relies on the recollection of a large number of changing camera views and scenes, which adds an additional burden to the research work. This paper intends to explore a new exploration approach based on the existing dataset itself. Regarding the differences in domain distributions, Guo et al.^46^ proposed an instance-specific batch normalization (IsBN) module, which uses IsBN as the base modulator to adjust the information flow to adapt to different domain distributions. It has good performance in handling multi-domain crowd counting and multi-domain learning. From the perspective of domain adaptation, the continuous feature transfer of source domain data to the target domain has received a lot of attention recently^47–50^. Zhou et al.^51^ conducted a comprehensive review on domain generalization problems, fully elaborating on the impact of data distribution differences or environmental changes on algorithms and key points of concern. Dividing a domain into dataset level and image level is a novel guided learning strategy^52^, which is conducive to a single model to adaptively process multi-domain datasets.

Single source domain generalization is a specific scenario and a challenging problem in domain generalization. Currently, there is not much work being done in this area, and it is still in the early stages of exploration. Single source domain generalization aims to train a model that can generalize to multiple unseen target domains^53^. A study has proposed a dynamic subdomain partitioning scheme on a single domain, dividing the source domain into multiple subdomains^54^, thereby creating a meta learning framework for domain generalization. This enhances the domain invariant and domain specific memory modules to re-encode image features, making the model have strong generalization ability. Regarding the issue of domain bias, Peng et al.^55^ proposed an MPCount method, which achieves feature invariant reconstruction through a memory bank and content error mask, and then uses image block matching to calculate classification loss, improving the accuracy of single domain generalization crowd counting. In order to overcome the domain gap,^56^ used the Dual Domain Teacher (D3T) framework to separate the source training set and the target training set to construct a dual teacher model, which was successfully applied to the student model to learn from each domain’s teacher model separately. These works provide a research foundation for single domain generalization. However, there is still considerable scope for further research, mainly manifested in poor performance on other unseen datasets. Additionally, achieving consistency in performance across multiple datasets remains an ongoing challenge.

## Methodology

**Fig. 2:**
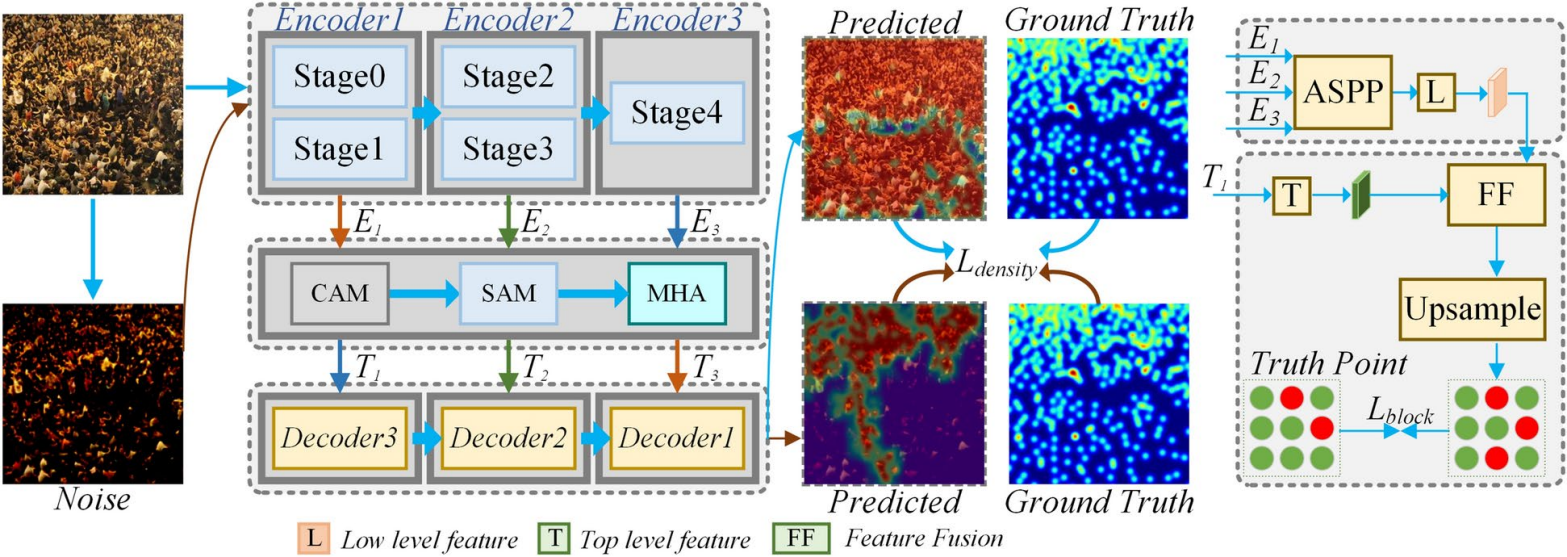
The overall architecture of MAHE.

The overall architecture of the proposed MAHE (Multi-scale Attention and Hierarchy level Enhancement) is shown in Fig. 2. The backbone network adopts the overall structure of Resnet50, which mainly consists of five stages. Stage0 is the preprocessing of input, and the last four stages are composed of Bottleneck, with similar structures. Stage1 contains 3 Bottlenecks, while the remaining three stages consist of 4, 6, and 3 Bottlenecks respectively. Set Stage0 and Stage1 as the first encoding layer, Stage2 and Stage3 as the second encoding layer, and Stage4 as the third encoding layer. These three encoding layers provide a foundation for subsequent multi-scale feature information mining calculations.

In the encoding layer of the model, Resnet is divided into three parts, with Stage0 and Stage1 as the first encoding layer Encoder1, Stage2 and Stage3 as the second encoding layer Encoder2, and Stage4 as the third encoding layer Encoder3. For an image with a width and height of H $\times$ W, the output width and height of Encoder 1 are H/4 $\times$ W/4, Encoder 2 is H/16 $\times$ W/16, and Encoder 3 is H/32 $\times$ W/32.

In the decoding process, convolution, two-dimensional batch normalization (BatchNorm2d), and ReLU were used. BatchNorm2d normalizes input data by converting features of different scales into the same scale, improving the model’s generalization capacity, accelerating model training, and preventing gradient explosion. And divide the decoding layer into three stages (Decoder 1, Decoder2, Decoder3), Each decoding layer corresponds to an encoding layer.

In Fig. 2, the feature $E_1$ extracted by the encoding layer Encoder1 composed of Stage0 and Stage1 is processed by the attention mechanism module to extract feature $T_3$, which does not include MHA processing. The feature $E_2$ extracted by the encoding layer Encoder2, which combines Stage2 and Stage3, is processed by the attention mechanism module to extract the feature $T_2$. The feature $E_3$ extracted by the encoding layer Encoder3 constructed by Stage4 is processed by the attention mechanism module to extract the feature $T_1$. The final decoding layer Decoder1 outputs a density map, which is used in the training stage to compare with the real density map for corresponding loss calculation.

In the model, pre-trained backbone networks (such as Resnet-50) are used to extract image features. To enhance the model’s ability to express features, feature enhancement is performed through the use of channel attention features and spatial attention features. Considering the shortcomings of crowd density analysis in terms of global feature confidence and regression capacity, a block matching mode based on ASPP multi-scale feature fusion after encoding is proposed to improve the stability and accuracy of the model.

In response to the problem of reduced feature information expression capability when the last two layers of the three-stage encoding layer are at a low scale, this paper adopts multi-head attention to further enhance the feature description capability of the current stage, which complements and promotes the encoding capability of the entire model.

In order to effectively improve the domain generalization capacity of the model, the lower branch of the model performs appropriate operations on the input data, so that there is a significant spatial structural difference between the input data and the upper branch. However, the position information of the research object remains unchanged, and the main frequency domain component information remains unchanged. The objective is to ensure that the model performs well on untrained datasets.

The encoded features are upsampled by the ASPP multi-scale feature module, thereby obtaining the classification feature map of the block. The ASPP multi-scale feature module can process the feature expressions of different blocks in the image more finely, which is beneficial for improving the model’s understanding of important feature information during the training stage, approaching key regional features, and ultimately obtaining an accurate crowd estimation model.

### Spatial self-attention feature extraction

**Fig. 3:**
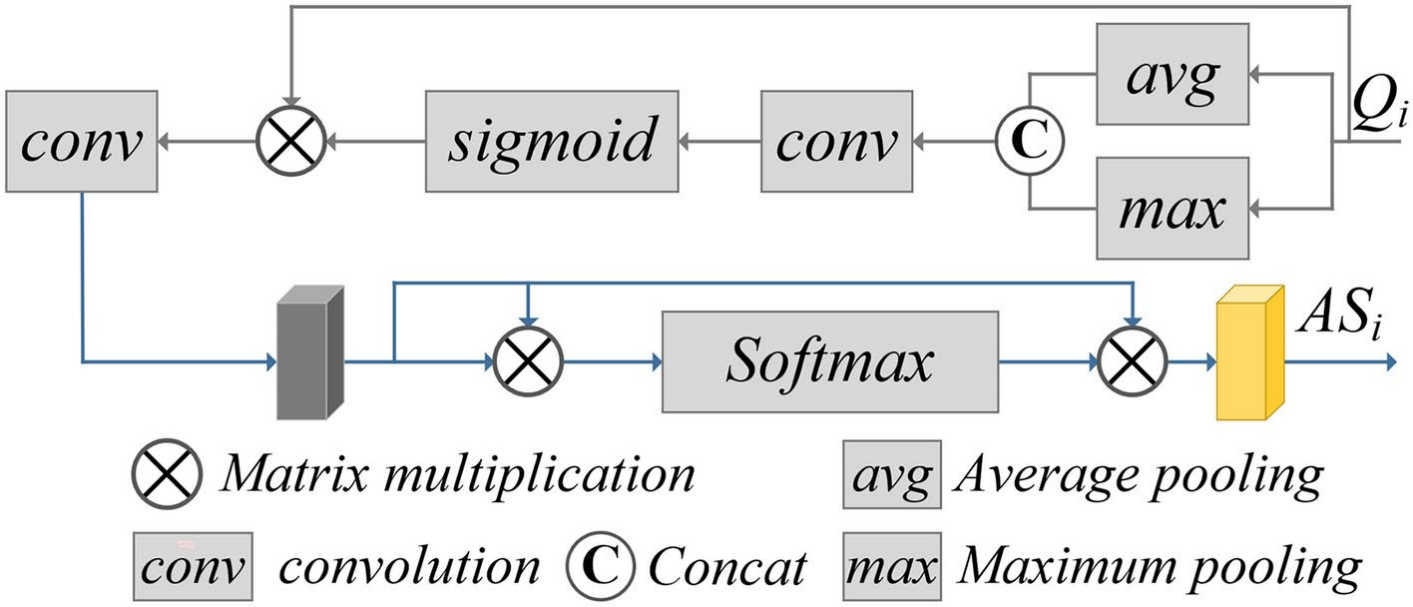
Simplified spatial attention module.

An input image $I\in R^{C\times H\times W}$,obtain corresponding outputs at different encoding layers $E_1\in R^{C\times \frac{H}{4}\times \frac{W}{4}}$, $E_2\in R^{C\times \frac{H}{16}\times \frac{W}{16}}$, $E_3\in R^{C\times \frac{H}{32}\times \frac{W}{32}}$, we designed a simplified version of the Spatial Attention Module (SAM) to effectively refine features (Fig. 3). Here, $\textcircled {x}$ represents matrix multiplication operation, $\textcircled {c}$ represents matrix connection operation in the channel direction, *conv* represents convolution operation, *avg* represents average pooling operation, and *max* represents max pooling operation. We first use the average avg and maximum max operations along the channel axis to generate two different single channel spatial maps and convert them into $\copyright$ Connect them together and calculate the spatial attention map using a 3$\times$3 convolutional conv and sigmoid function. The spatial attention map reweights the features from the spatial dimension by element wise multiplication, and the refined weighted features are fed into a 3$\times$3 convolutional layer conv to compress the channels to the required number.

In order to effectively focus on spatial features and simplify calculations, a simplified self-attention method is used to extract spatial attention features at different scales. To capture semantic information related to the head of a crowd in depth, a multi head attention mechanism is adopted, let the number of long positions be h, initial $Q=K=V=E_i,(i\in [1,2,3])$,then the spatial attention features $AS_i$ of the corresponding encoding layer $E_i$ is $AS_i=MH(Q,K,V)$, where MH represents multi head attention, and it can be expressed as shown in the following equation.

1 $$\begin{aligned} & MH\left( Q,K,V\right) =Concat\left( {head}_1,{head}_2,\cdots ,{head}_n\right) W \end{aligned}$$

2 $$\begin{aligned} & {head}_j=Dropout\left( Softmax\left( \frac{Q_jK_j}{\sqrt{\frac{d_{model}}{h}}}\right) \right) V_j \end{aligned}$$

Wherein, $W\in \ R^{d_{model}\times d_{model}}$is the output projection matrix, $d_{model}$ is the dimension of attention, the dropout function plays a role in preventing overfitting and improving the model’s generalization capacity. Due to the upper limit of the number n of heads in this paper is 8, the range of j in Eq. (2) is j $\in$[1,2,...,8].

Using the Feature Pyramid Network (FPN) as a reference to implement multi-scale feature upsampling calculations, except for the last layer Encoder 1 which directly decodes, the subsequent decoding layers Decoder 2 and Decoder 3 both require upsampling and spatial self-attention features to be combined and passed to the next layer.

### Channel self-attention feature extraction

**Fig. 4:**
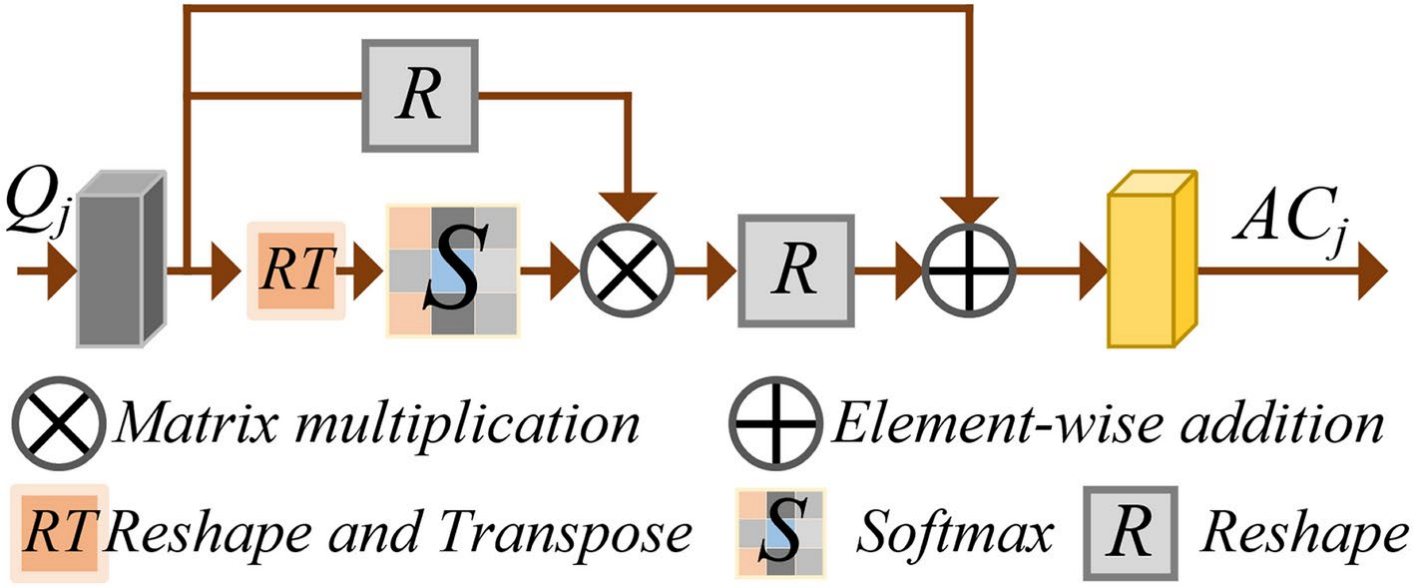
Channel attention module.

Unlike spatial self-attention multi-scale feature extraction, channel self-attention computation introduces Q-CAM (Channel Attention Module) computation, calculate the reshape of K represented by R in Fig. 4. Before extracting channel attention features, random image denoising and Gaussian blur processing were performed to enhance the model’s generalization capacity. The attention features of the encoding layer $E_i (i\in [1,2,3])$channel correspond to the QKV to be calculated, and $AC_j$ is inferred and calculated according to the following formula. Where RT means Reshape and Transpose operations, S means Softmax operations, and R means Reshape operations.

3 $$\begin{aligned} \text {AC}_j=R\left( Softmax\left( R\left( Q_j\right) T\left( R\left( K_j\right) \right) \right) R\left( V_j\right) \right) \times \gamma +Q_j \end{aligned}$$

In the above equation,firstly, the calculation of Query involves dimension transformation of Q, $R(Q_j )$ implementation transforms dimension B$\times$C$\times$H$\times$W to B$\times$C$\times$N, the calculation of Key includes dimension transformation $R\left( \cdot \right)$, and $T\left( \cdot \right)$, the function T implements two steps of dimension swapping from (B$\times$C$\times$N) to (B$\times$N$\times$C). Then multiply the obtained two feature maps Q by K, obtain the feature map $CAM\_S$ through Softmax again, its dimension is B$\times$C$\times$C. Finally, multiply $CAM\_S$ and $R(V_j)$ in a matrix, obtain output O, dimension is B$\times$C$\times$N. After $\textbf{O}$=R(O) operation, the dimension becomes the original dimension B$\times$C$\times$H$\times$W. Finally, multiply $\textbf{O}$ by the scale coefficient $\gamma$ and add $Q_j$ to obtain the final output $AC_j$. Its multi-scale calculations are consistent with 3.1, using the FPN calculation mode.

### Multi-scale hierarchy level feature fusion

The feature analysis process of encoding and decoding using Resnet as the backbone is effective in parsing high-level semantic information. When the crowd is abnormally crowded, the model also has a certain representation ability for low-level semantic details. However, it is difficult to parse high-level semantics and low-level semantic intervals from the near to the far. To overcome this problem, this work considers the correlation between the final output of the decoding layer and the final output of the encoding layer to improve the overall performance of the model.

In order to effectively achieve complementary Feature Fusion (FF) of features at different levels, it is necessary to fully consider the correlation between low-level feature maps and high-level feature maps, which is referred to as the correlation degree Corr of feature maps. The FF implementation relies on the ASPP (Atrous Spatial Pyramid Pooling) module, which consists of four parallel dilated convolution modules (with different convolution rates) to obtain a larger receptive field and extract more contextual information. If the i-th low-level feature map point is Li and the j-th high-level feature map point is Tj, then $Corr_{ij}$ is used to measure the correlation between the i-th position point in the low-level feature map and the j-th position point in the high-level feature map. In order to facilitate fusion computation of feature maps at different scales, FF Module is mainly constructed based on spatial attention mechanism. FFM achieves point correlation on different feature maps through batch matrix multiplication, and the correlation degree $Corr_{ij}$ is used for the weight vector of high-level feature maps.

4 $$\begin{aligned} \text {Corr}_{\text {ij}} = \frac{exp(L_i \cdot T_j)}{\begin{matrix} \sum _{i=1}^N {L_i \cdot T_j} \end{matrix}} \end{aligned}$$

**Fig. 5:**
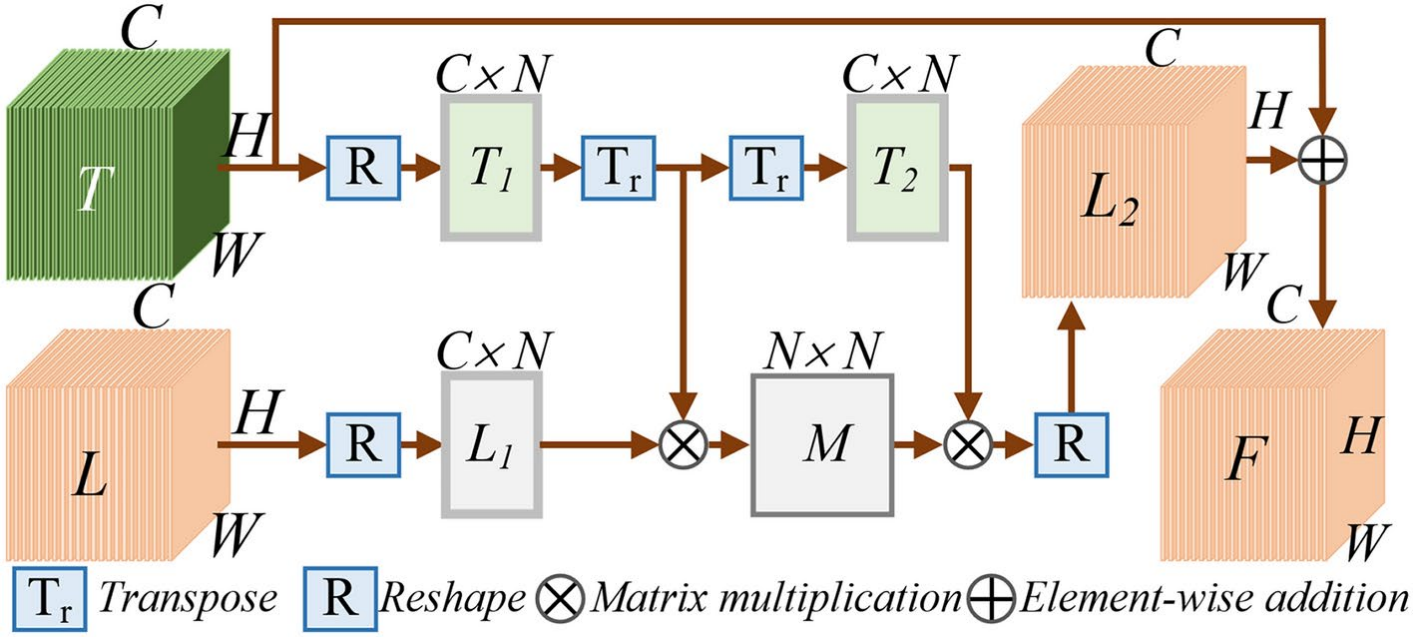
Feature fusion module.

In the above equation, L and T represent the low-level and high-level feature maps obtained point by point, N represents the number of points, corresponding $\left\{ L,T\right\} \in \ R^{C\times N}$, C is the number of channels, and their correlation is directly proportional to the similarity between two position points. FFM is shown in Fig. 5.

Before performing feature fusion, perform channel dimensionality reduction on the high-level feature map *T* and low-level feature map *L*, where $\left\{ T,L\right\} \in \ R^{C\times H\times W}$, *C* is the number of channels in the feature map, and *H* and *W* represent width and height. Then perform the same deformation operation on both the high-level feature map *T* and the low-level feature map *L* until $R^{C\times N}$ to obtain the corresponding $T_1$ and $L_1$,where N=H$\times$W, the attention feature map M $M\in \ R^{N\times N}$ is obtained by matrix multiplication of $T_1$ transpose and $L_1$, and $T_2$ is obtained by transpose of $T_1$ again. After Softmax calculation, $T_2$ and *M* are matrix multiplied and transformed to $L_2\in \ R^{C\times H\times W}$. Finally, a point by point addition operation is performed on the high-level feature maps *T* and $L_2$ to obtain the final fused feature output $F\in \ R^{C\times H\times W}$as follows:

5 $$\begin{aligned} F_i=\alpha \ R\left( \left( {T}_{\text {2i}}\times M\right) \right) +T_i \end{aligned}$$

In the above formula, $\alpha$ is the pre assigned weight for the association between high-level feature maps and low-level feature maps. Through model training and learning, $\alpha$ will receive more attention and continue to improve, while Fi represents the features fused on the i-th channel. The resulting fused feature map F is an output that fully considers the semantic information of high-level and low-level, highlighting the integration of low-level detail information into high-level global information, thereby improving the semantic feature extraction performance of the head boundary.

In order to fully explore low-level detail information, this paper conducted multi-scale feature fusion calculations for *L* feature maps at different scales (involving Decoder 1, Decoder 2, Decoder 3). The feature layers Decoder 2 and Decoder 3 need to be upsampled and calculated according to formulas 4 and 5.

### Block matching of semantic activation features

**Fig. 6:**
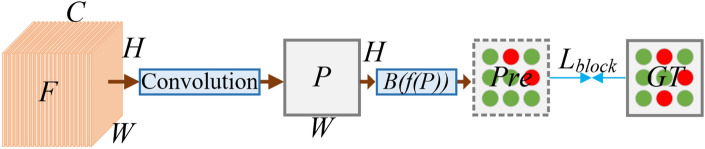
Block matching module.

The calculation process of module BMSA (Block Matching of Semantic Activation features) is shown in Fig. 6. The feature map F is convolved to obtain a feature map P with a size of H$\times$W. The activation calculation of P is performed, and if the threshold $\epsilon$ is exceeded, it is assigned a value of 1, otherwise, it is set to 0. Then, block operation is performed to divide P into blocks with a size of 32$\times$32. If the size is less than 32, a zero-padding operation is performed. The predicted classification result Pre is $R^{\frac{H}{32}\times \frac{W}{32}}$, and the total number of blocks is B. If other smaller blocks are used, the corresponding Pre needs to be upsampled. The ground truth (GT in Fig. 6) is also calculated in blocks according to this operation, and finally $L_\text {block}$ is calculated using binary cross entropy loss, as shown in Eq. (6).

6 $$\begin{aligned} L_\text {block}=-\frac{1}{B}\begin{matrix}\sum \nolimits _{i=1}^B{GT_i \log (pre_i)+(1-GT_i) \cdot \log (1-pre_i)}\end{matrix} \end{aligned}$$

Where B is the number of blocks, $pre_i$ is the predicted value of the i-th block, $GT_i$ is the ground truth value of the i-th block, and log($\cdot$) is the natural logarithm function.

### Loss function

When calculating the training error of the model, the global and local correlations between the density map and the real density map are combined, and the classification consistency of multi-scale blocks is combined with Euclidean loss to effectively measure the consistency estimation between global and detail. The density map loss is calculated using RMSE loss, as shown in Eq. (7).

7 $$\begin{aligned} L_\text {density}=\sqrt{\frac{1}{N}\begin{matrix}\sum \nolimits _{i=1}^N \left| d_i-d_i^{gt} \right| ^2 \end{matrix}} \end{aligned}$$

Where N is the number of points in the density map, di is the predicted i-th density value, and $d_i^{gt}$ is the i-th true density value in the actual density map. The final objective function is obtained by weighting and summing the two loss functions $L_{block}$ and $L_{density}$ mentioned above, and the entire network is trained using the following objective function.

8 $$\begin{aligned} L=\lambda _1L_{s\_{density}}+\lambda _2L_{s\_{block}}+\lambda _3L_{c\_{density}}+\lambda _4L_{c\_{block}} \end{aligned}$$

Among them, $L_{s\_{density}}$ represents the density map loss under the spatial attention module, $L_{s\_{block}}$ represents the semantic feature block matching loss under the spatial attention module, $L_{c\_{density}}$ represents the density map loss under the channel attention module, $L_{c\_{block}}$ represents the semantic feature block matching loss under the channel attention module, and $\lambda _i$ is the corresponding loss coefficient.

## Experiment

In order to train and evaluate our proposed crowd counting model, we selected three widely used crowd counting datasets, ShanghaiTech-A/B^57^, UCF-QNRF^58^ and JHU Crowd++^59^. ShanghaiTech: Contains 1198 annotated images with a total of 330165 people, divided into two parts: A and B. A contains 482 images, all of which are downloaded from the internet and contain highly crowded scene images with crowd sizes ranging from 33 to 3139. The training set contains 300 images and the testing set contains 182 images. B contains 716 images with relatively sparse pedestrian flow scenes, captured by fixed cameras on the streets, with groups ranging from 12 to 578. The training set contains 400 images and the test set contains 316 images. UCF-QNRF: it contains 1535 dense crowd images from Flickr, web searches, and Hajj clips. The dataset includes a wide range of scenes with rich perspectives, lighting variations, and density diversity. The counting range ranges from 49 to 12865, making the database more difficult and realistic. In addition, the image resolution is also very high, resulting in significant changes in head size.

JHU Crowd++ is a large-scale crowd counting dataset containing 4372 images, with training, validation, and testing samples of 2272, 500, and 1600, respectively. In this experiment, 879 images annotated as “Stadium” (SD) and 572 images annotated as “Street” (ST) were selected from the dataset as the source domains for training and testing. The ratio of the training set, validation set, and test set was set to 6:1:3.

### Experimental environment and training details

This experiment was conducted on a server equipped with NVIDIA 4090 GPU, using Cuda 12.4, PyTorch 2.3, and Python 3.10. In the preprocessing stage of model training data, the input training samples were randomly cropped into images with a size of 320$\times$320. If the short side of the original image is less than 320, an enlargement operation was performed. To ensure that the input samples have good generalization capacity in the spatial attention branch and channel attention branch, different image preprocessing operations were performed in different branches to enhance the system’s adaptability, such as random flipping, random scale changes (0.8 to 1.2 times), random lighting enhancement, random blurring, and other operations on the input image. In addition, random image jitter enhancement was performed to increase data diversity and better adapt to different environments. During the training process, the Adamw optimizer was used, with an initial learning rate of 1e-3 and 240 training iterations. The learning rate adjustment strategy adopted the OneCycleLR method, and the corresponding parameter $final\_div\_factor$ was set to 1e3. Adopting this learning mechanism can effectively improve the stability, consistency, and generalization capacity of optimization algorithms, enabling them to perform better on unseen data.

### Evaluating indicator

**Table 1:** Comparison with the state-of-the-art methods on SHA (A), SHB (B) and UCF-QNRF (Q).

Method	$A \rightarrow B$		$A \rightarrow Q$		$B \rightarrow A$		$B \rightarrow Q$		$Q \rightarrow A$		$Q \rightarrow B$	
	MAE	RMSE	MAE	RMSE	MAE	RMSE	MAE	RMSE	MAE	RMSE	MAE	RMSE
DMCount^60^	23.1	34.9	134.4	252.1	143.9	239.6	203.0	386.1	73.4	135.1	14.3	27.5
SASNet^61^	21.3	33.2	211.2	418.6	132.4	225.6	273.5	481.3	73.9	116.4	13.0	22.1
MAN^62^	22.1	32.8	138.8	266.3	133.6	255.6	209.4	378.8	67.1	122.1	12.5	22.2
RBT^63^	13.4	29.3	175.0	294.8	112.2	218.2	211.3	381.9	-	-	-	-
C2MoT^64^	12.4	21.1	125.7	218.3	120.7	192.0	198.9	368.0	-	-	-	-
DCCUS^54^	12.6	24.6	119.4	216.6	121.8	203.1	179.1	316.2	67.4	112.8	**12.1**	20.9
MPCount^55^	**11.4**	19.7	**115.7**	**199.8**	**99.6**	182.9	165.6	290.4	**65.5**	110.1	12.3	24.1
MAHE (Ours)	12.1	**19.5**	118.1	203.5	103.6	**178.7**	**162.5**	**285.8**	67.3	**108.7**	12.2	**20.7**

Most studies in the field of crowd density estimation use Mean Absolute Error (MAE) and Root Mean Square Error (RMSE) as evaluation metrics. In order to conduct experimental comparative analysis with previously published evaluation data, we also use mean absolute error and root mean square error as evaluation indicators. MAE reflects the error between the predicted number of human heads through the model and the actual number of human heads in the image, while RMSE describes the degree of difference between the predicted number of human heads through the model and the true number of human heads in the image, which are defined as:

9 $$\begin{aligned} & MAE=\frac{1}{N}\begin{matrix}\sum \nolimits _{i=1}^N \left| c_i-c_i^{GT} \right| \end{matrix} \end{aligned}$$

10 $$\begin{aligned} & RMSE=\sqrt{\frac{1}{N}\begin{matrix}\sum \nolimits _{i=1}^N \left| c_i-c_i^{GT} \right| ^2 \end{matrix}} \end{aligned}$$

Where N is the number of test images, $c_i$ is the predicted number of people in the i-th test image, and $c_i^{GT}$ is the actual number of people in the i-th test image.

### Comparison with State of the Art

We conducted experiments through SHA, SHB, QNRF training, and interactive testing. By training on the source domain and testing on the target domain, the effectiveness of the algorithm proposed in this paper was evaluated, and the effectiveness of the model inference scheme was verified. Compared with MPCount and DCCUS, everyone has their own advantages, compared with other methods, the method proposed in this paper performs very well. Overall, our method is superior to other state-of-the-art technologies to a certain extent. Some methods may look good in certain settings, but they are poor in other task settings, indicating that they do not perform well in multiple domains and their generality is not very good.

In most of these settings, our MAHE has significant advantages in domain generalization methods, including significant reductions in both B$\rightarrow$A and B$\rightarrow$Q. The experimental results are shown in Table 1.

**Table 2:** Comparison with the state of the art on data in JHU-Crowd++ with labels “Stadium”(SD), “Street”(SR).

Method	$SD \rightarrow SR$		$SR \rightarrow SD$	
	MAE	RMSE	MAE	RMSE
MAN^62^	45.6	79.5	247.3	954.7
DCCUS^54^	90.2	193.6	257.8	1004.3
MPCount^55^	**39.1**	71.4	219.4	937.2
MAHE (Ours)	41.9	**68.7**	**216.1**	**782.0**

In Table 2, we further conducted experiments on specific scene domain SD $\leftrightarrow$ SR. MAN^62^, DCCUS^54^, and MPCount^55^ used their published code for training, and the results showed that these algorithms were difficult to achieve good performance when the source domain data distribution was narrow. Under the same conditions, our MAHE performs the best in the RMSE metric, and in the MAE metric, MAHE is comparable to MPCount.

### Ablation studies

Each important component in the module is crucial for the performance of the entire model. To facilitate the description of ablation experiments, B represents Baseline, UB represents Upper Branch, LB represents Lower Branch, C represents Classification, IP represents Image Preprocessing, FF represents Feature Fusion, and N represents Noise. The model considers factors such as feature extraction capability, multi-scale feature fusion, and feature transfer integration.

Firstly, placing the IP at the beginning of the input module can help improve the adaptability of the model, especially the processing of different IP addresses in the upper and lower branches can enhance the model’s generalization ability on different data domains. The channel and spatial attention enhancement module ensures that the model can effectively extract feature information while maintaining control over the model size, and is therefore directly embedded as an important component of the basic model. The LLF model helps to enhance feature fusion at multiple scales, which can further improve the feature transfer performance of the model.

**Fig. 7:**
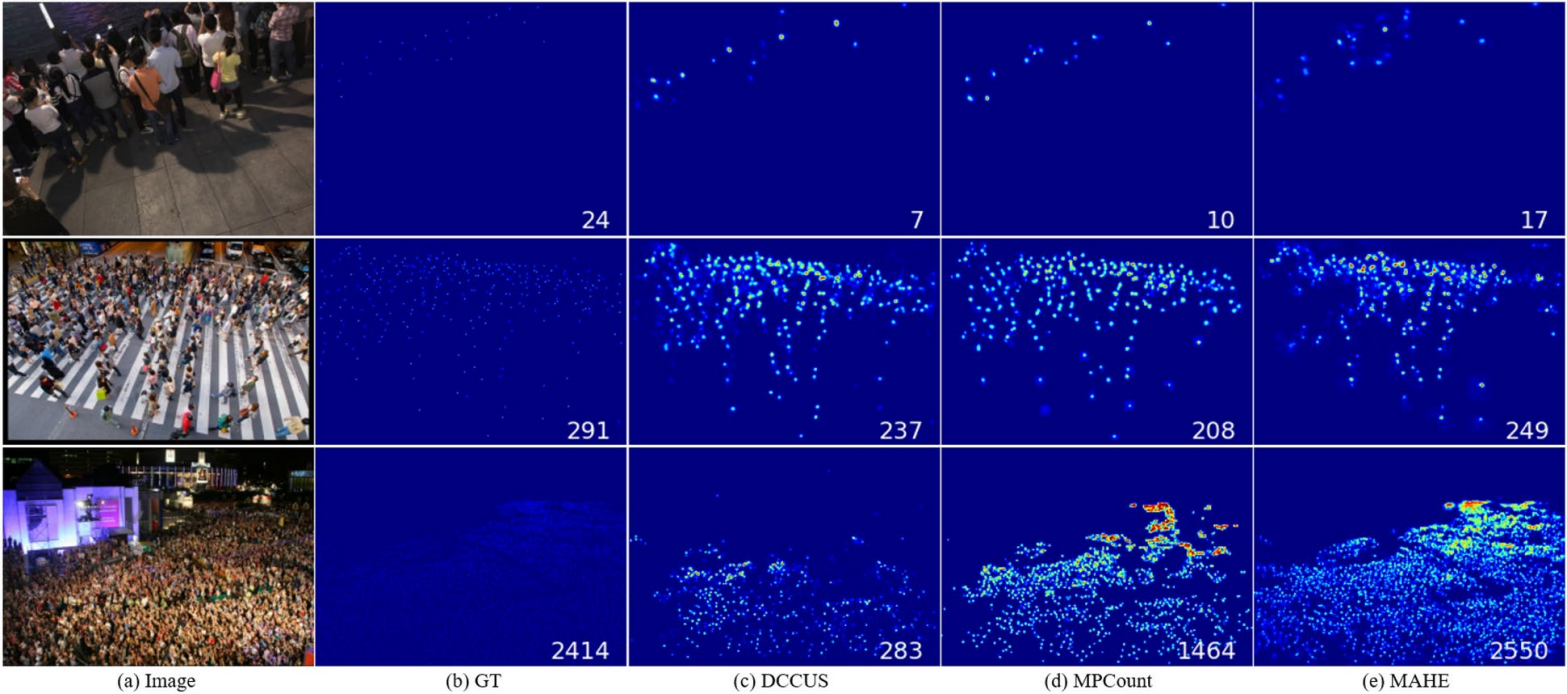
Visualization results of DCCUS, MPCount and MAHE under different DG settings. First row: A$\rightarrow$B; second row: A$\rightarrow$Q; third row: B$\rightarrow$Q.

In this ablation experiment, we aim to investigate the specific effects of different combinations of component blocks on model performance. We trained the proposed MAHE model on the SHA dataset and tested it on the SHB dataset for a series of ablation experiments. We conducted ablation studies on $SHA \rightarrow SHB$. In the experiment, we changed different combinations to observe how these changes affect the changes in MAE and RMSE of the model. By comparing different block combinations, we found that although all combinations were not optimal in terms of parameter and computational complexity, they performed the best in MAE and RMSE. This indicates that differentiated preprocessing and sufficient feature acquisition can more effectively capture crowd density feature descriptions, thereby improving the predictive performance of the model. The data in Table 3 further confirms this conclusion.

**Table 3:** Ablation study on our proposed components.

No.	B	UB	LB	C	IP	FF	N	MAE	RMSE
1	$\checkmark$	$\checkmark$	-	$\checkmark$	-	-	-	19.0	28.4
2	$\checkmark$	-	$\checkmark$	$\checkmark$	-	-	-	19.1	28.6
3	$\checkmark$	$\checkmark$	$\checkmark$	-	-	-	-	16.9	27.3
4	$\checkmark$	$\checkmark$	$\checkmark$	$\checkmark$	-	-	-	13.7	21.8
5	$\checkmark$	$\checkmark$	$\checkmark$	$\checkmark$	$\checkmark$	-	-	12.9	20.1
6	$\checkmark$	$\checkmark$	$\checkmark$	$\checkmark$ $\checkmark$	$\checkmark$	$\checkmark$	-	12.4	19.6
7	$\checkmark$	$\checkmark$	$\checkmark$	$\checkmark$	$\checkmark$	$\checkmark$	$\checkmark$	12.3	19.5

B(Baseline)+UB(Upper Branch). Our baseline consists only of the basic encoder decoder and density estimator. In all tests, we applied Channel and Spatial Attention Module (CSAM) to the baseline. CSAM extracts channel attention and spatial attention features, and combines them with encoding features to enhance the decoding output capability of the model. This greatly improves all the experiments in Table 3. Not using CSAM will increase the overall errors of MAE and RMSE by more than 2 percentage points, indicating the necessity of using CSAM technology for feature enhancement evaluation.

B+LB(Lower Branch). Comparing B+UB and B+LB, we found that independently applying a certain branch has little impact on the model, and both can independently complete crowd counting, but the performance is not good, with a MAE of over 19.

B+UB+LB+C(Classification). We have added a classification loss module to both the upper and lower branches, which has a good promoting effect on the model. Without the classification loss module, the performance will be greatly reduced.

IP(Image Preprocessing). We found that the dual branch structure of the model is very important for image preprocessing in the input stage. Without adding preprocessing operations, the model will degrade. Therefore, appropriate IP operations are closely related to the final generalization ability of the model, which currently involves color enhancement, flipping, rotation, translation, and other operations.

**Fig. 8:**
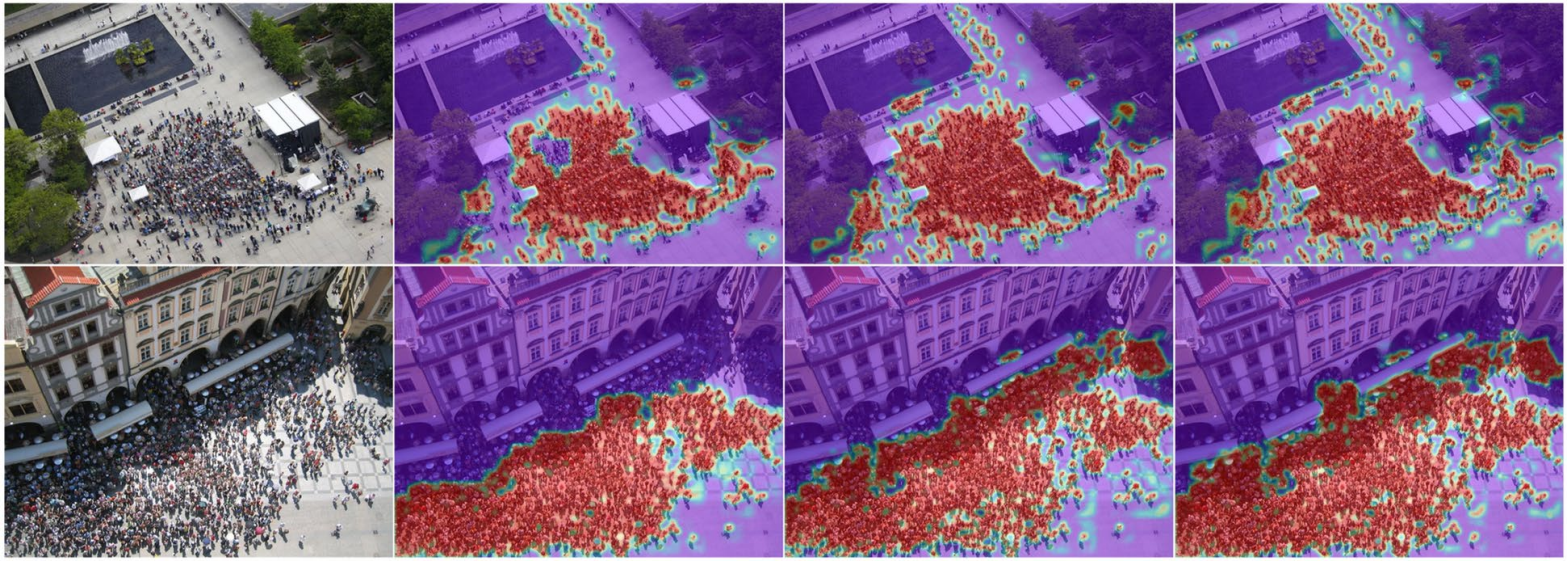
Visualization of MAHE model inference optimized by different components.

W/O FF(Feature Fusion). When calculating classification loss, if FF is not added, the effect will slightly decrease. This indicates that after FF fusion of top-level features and low-level features, the classification features of the model are enhanced, which verifies the effectiveness of the encoder in re encoding image classification features. The multi-scale feature fusion of ASPP has a good complementary ability for this model in different encoding layers, and can fully explore the detailed feature information of different encoding layers.

W/O noise. In the image preprocessing stage of the lower branch, adding noise and not adding noise have a certain impact on the overall generalization performance of the model. The experimental results are shown in Table 3.

### Experimental comparison visualization effect

In this section, we compare MAHE with the latest methods on different benchmarks, as shown in Fig. 7. The selected baselines can be divided into three categories: those with a crowd size of less than 500, those with a crowd size of less than 1000, and those with a crowd size exceeding 4000. The results of A$\rightarrow$B, A$\rightarrow$Q, and B$\rightarrow$Q are shown. We downloaded the source code and directly tested the model from the publicly available GitHub website of the paper.

In Fig. 7, the visualization results of DCCUS, MPCount, and MAHE under different DG settings are shown. First line: A$\rightarrow$B; Second line: A$\rightarrow$Q; The third line: B$\rightarrow$Q. The real number of people in the first row is 24, and the real number of people in the second row is 291. The MAHE algorithm has similar inference results, showing good generalization ability. In the third row, the inference effects of multiple algorithms are not ideal, with significant differences. This indicates that there is still a lot of room for model optimization when the crowd size is particularly large.

### MAHE model visualization

The following figure shows the visualization effects of different model parameters trained on A and tested on Q. The basic model using Resnet50 encoding and decoding, combined with channel and spatial attention models, includes a multi head attention model with all components. It can be seen that the final model has good performance.

In Fig. 8, the first column is the original image of the Q dataset, the second column is the inference result of the Resnet50 encoding and decoding base model, the third column is the inference result of the base model combined with channel and spatial attention, and the fourth column is the final inference effect of the multi head attention model containing all components.

## Conclusion

The crowd counting framework MAHE proposed in this paper significantly improves the recognition accuracy and robustness of single domain generalized crowd counting through a series of innovative techniques. Firstly, by introducing channel attention and spatial attention, the model not only pays attention to the detailed features of each channel, but also considers the positional changes in the spatial structure, improving the efficient feature acquisition ability of the model. Secondly, the addition of multi-head attention feature module enables the model to effectively capture complex dependency relationships between sequence elements. In addition, the three-stage encoding and decoding processing mode enables the model to effectively represent crowd density information. This paper further enhances the fusion of multi-scale features of different receptive fields through Multi-scale Hierarchy Level Feature Fusion, MHLFF, to ensure that the model learns high-level semantic information and low-level multi-scale visual feature information. On the relevant ShanghaiTech and UCF-QNRF datasets, it has been experimentally verified that excellent performance has been achieved in single domain generalization of crowd counting tasks, especially in challenging scenarios such as low-density and high-density crowd, where the performance advantage is more pronounced. However, testing on specific datasets may not fully cover the diversity in real-world scenarios, such as models that still need improvement in handling occlusion, adverse weather, and complex backgrounds. Future work will focus on improving the generalization ability of the model through more comprehensive feature representation algorithms and more reasonable differentiation branch loss calculations, further optimizing computational efficiency, and exploring more robust crowd counting methods.

## Data Availability

The datasets used or analysed during the current study available from the corresponding author on reasonable request.
